# Reply to Vanunu and Newell: The frequent-winner effect is necessary to explain experience-based decisions

**DOI:** 10.1073/pnas.2500422122

**Published:** 2025-04-07

**Authors:** Sebastian Olschewski, Mikhail S. Spektor, Gaël Le Mens

**Affiliations:** ^a^Department of Psychology, University of Basel, Basel 4055, Switzerland; ^b^Warwick Business School, University of Warwick, Coventry CV4 7EQ, United Kingdom; ^c^College of Arts and Sciences, VinUniversity, Hanoi, Vietnam; ^d^Department of Psychology, University of Warwick, Coventry CV4 7AL, United Kingdom; ^e^Department of Economics and Business, Universitat Pompeu Fabra, Barcelona 08005, Spain; ^f^Universitat Pompeu Fabra–Barcelona School of Management, Barcelona 08008, Spain; ^g^Barcelona School of Economics, Barcelona 08005, Spain

What attitudes do people have toward the skewness of outcome distributions, and why? Our recent publication (1) reconciled seemingly contradictory observations: Preferences for right-skewed distributions in financial decisions in the wild (2, 3) and in various experimental tasks (4–6) and preferences for left-skewed distributions in laboratory experience–based binary decisions (7, 8). We found that when people engage in an outcome-comparison process, they tend to choose the option that results in better outcomes most of the time (*frequent-winner effect*). This is typically the left-skewed option, as it produces many outcomes above its mean and few extreme outcomes below it. When no frequent-winner option is present, people tend to choose right-skewed outcome distributions, in line with previous findings in other settings (2–6). We attributed this tendency to intrinsic preferences in favor of right-skewed outcome distributions.

In their comment, Vanunu and Newell (9) proposed an alternative mechanism for why people might choose the right-skewed option when there is no frequent winner in our study. Specifically, they argued that participants might make decisions by focusing on a small subset of observed outcome pairs, those with the largest absolute differences (10). To support this hypothesis, they reanalyzed the outcomes presented in our Study 3 (1) in which there was no frequent winner. They showed that, assuming that people draw mental samples of outcome pairs sorted from the largest to smallest absolute differences, they choose the right-skewed option only when relying on a small sample (1 to 9 accumulated ordered outcome pairs). In contrast, larger sample sizes favor the left-skewed option (10 to 29 accumulated ordered outcome pairs).

While a mechanism in which people rely on a small sample of ordered pairs is a promising attempt to reconcile the co-occurrence of choice tendencies for right- and left-skewed options when there is no frequent winner, we want to highlight an important limitation of this mechanism: It implies a choice pattern inconsistent with those observed in other conditions of our studies (1). For example, a decision-maker focusing on a small number of ordered pairs would choose the right-skewed option over the left-skewed option even in scenarios where the left-skewed option is the frequent winner (Fig. 1*A*). Yet, we found the opposite pattern: People were more likely to choose the left-skewed option, in line with the frequent-winner effect. The proposed mechanism also implies that participants should favor the option providing the smaller outcome most frequently in our condition with two identical Gaussian distributions (Fig. 1*B*). However, this also contradicts the empirical evidence consistent with the frequent-winner effect.

**Fig. 1.**
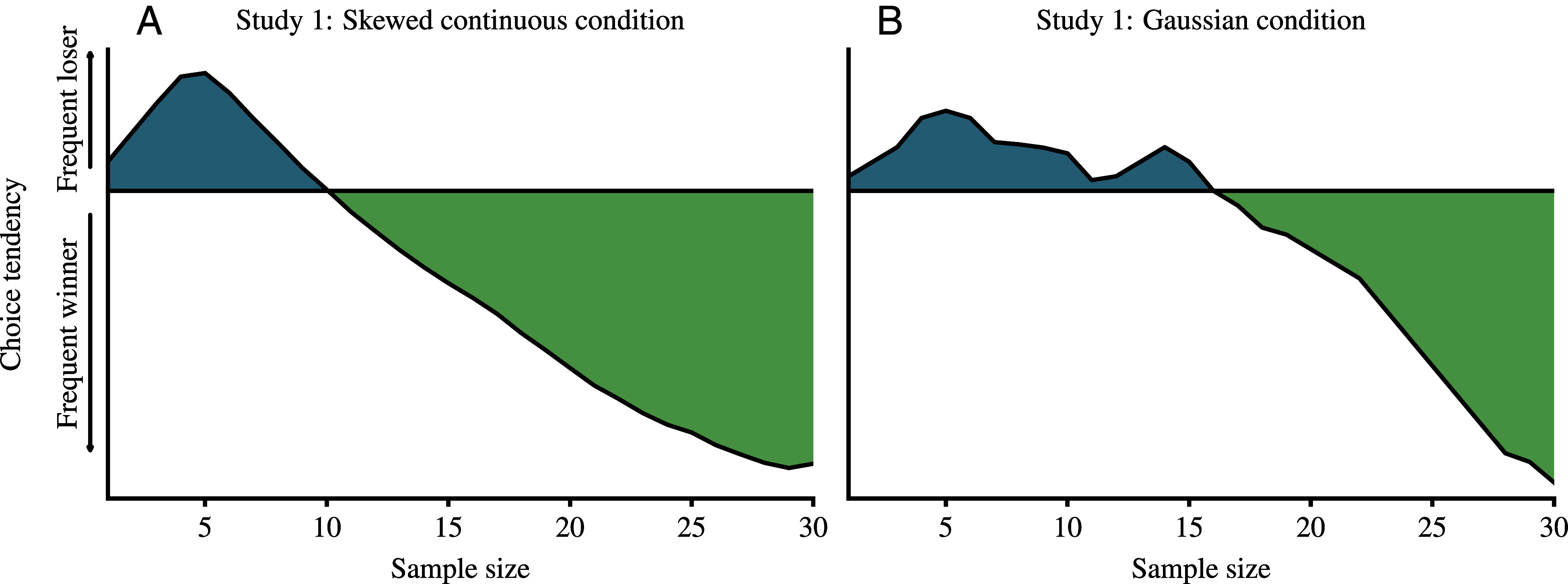
Visualizations of the predictions of the mechanism illustrated in figure 1 in ref. 9 using data from left- and right-skewed outcome distributions (*A*) and from two identical Gaussian distributions with a frequent winner induced through an experimental manipulation (20 out of 30 outcome pairs) (*B*) in Study 1 in ref. 1.

In summary, the hypothesis advanced in ref. 9 is based on a plausible cognitive process. However, its explanatory power is limited to situations where both options have an equal frequency of winning. Consequently, the frequent-winner effect remains an indispensable learning mechanism for explaining experience-based decisions and should be incorporated into future theorizing and modeling efforts. At the same time, we recognize the importance for future research to try to understand the stability of skewness effects when there is no clear frequent-winner option.

## Data Availability

The analysis code to reproduce the presented results and figures can be found under ref. 11.
